# Artificial Intelligence computed tomography models for the discrimination of Wilms versus non-Wilms tumors: systematic review and meta-analysis

**DOI:** 10.1590/2175-8239-JBN-2025-0010en

**Published:** 2025-10-10

**Authors:** Helvécio Neves Feitosa, Rian Vilar Lima, João Filipe Cavalcante Uchoa Furtado, Victor Arthur Ohannesian, Eduardo Correia Eulálio, Pedro Vianna Caldas Ribeiro, Lucas Macêdo Aurélio Paiva, Isabela Diógenes Feitosa, Geraldo Bezerra da Silva

**Affiliations:** 1 Universidade de Fortaleza, Escola de Medicina, Fortaleza, CE, Brazil.; 2 Faculdade de Ciências da Saúde Albert Einstein, Escola de Medicina, São Paulo, SP, Brazil.; 3 Universidade de Fortaleza, Escola de Medicina, Programas de Pós-Graduação em Saúde Pública e Ciências Médicas, Fortaleza, CE, Brazil.

**Keywords:** Artificial Intelligence, Machine Learning Algorithms, Meta-Analysis, Wilms Tumor

## Abstract

**Objective:**

To conduct a systematic review and meta-analysis to evaluate the effectiveness of artificial intelligence (AI) models aimed at identify Wilms tumor on computed tomography (CT) scans.

**Methods:**

A search was carried out across MEDLINE, Embase, Web of Science, and Cochrane databases in accordance with the Preferred Reporting Items for Systematic Reviews and Meta-Analyses (PRISMA) 2020 guidelines. Diagnostic studies using AI-based CT to diagnose Wilms tumor were included if they reported sensitivity, specificity, and AUC. Studies with incomplete data or lacking full-text availability were excluded. Statistical analysis was conducted in R (v4.3.3) using a random-effects model, with logit transformation for univariate analysis and SROC curve construction for bivariate analysis. Heterogeneity (I^2^ ≥ 40%) was assessed and explored via sensitivity analysis.

**Results:**

The analysis included four studies (three studies from China and one from Turkey) with 177 patients with Wilms tumors and 62 without Wilms tumors. The combined analysis of all models demonstrated a sensitivity of 63.9% (95% CI: 0.533–0.734), a specificity of 82.8% (95% CI: 0.716–0.902), and an area under the curve (AUC) of 0.831 (95% CI: 0.607–0.883).

**Conclusion:**

This study demonstrated that AI models exhibit moderate sensitivity and high specificity to identify Wilms tumor on CT scans, with an overall AUC of 0.831. These results underscore the promise of AI as a supportive tool in diagnostic imaging, although the limited number of studies and notable methodological heterogeneity warrant cautious interpretation and reinforce the need for validation in larger, more representative populations.

## Introduction

Wilms tumor, also referred to as nephroblastoma, is the most common form of kidney cancer in children, accounting for approximately 95% of pediatric renal tumors^1^. This malignancy is associated with mutations in critical genes such as WT1 and epigenetic alterations at specific loci, such as 11p15^2^. Although it generally has an excellent prognosis, with cure rates exceeding 90% in localized cases, identifying genetic and molecular factors remains essential for personalized treatment^3^. Clinical management relies on a collaborative effort among pediatric oncologists, surgeons, and pathologists, who apply specific protocols to stratify each patient’s risk^4^.

The application of artificial intelligence (AI) shows considerable promise in the diagnosis and management of renal tumors, including Wilms tumor^5^. Deep learning algorithms can analyze computed tomography images with high precision, detecting subtle features that may be overlooked by human observers^6^. Furthermore, these tools assist in distinguishing between benign and malignant renal masses, optimizing clinical decision-making and reducing diagnostic errors^7^. Integrating radiological and genomic data using AI offers detailed insights into tumor evolution and behavior, enabling the personalization of therapeutic interventions^8^.

Despite its promising capabilities, the clinical application of AI in differentiating renal tumors still faces important challenges. Overlapping radiological features among various renal neoplasms—such as Wilms tumor, congenital mesoblastic nephroma, and other pediatric renal masses—can limit diagnostic specificity, even for advanced algorithms. Moreover, variability in imaging protocols, limited access to large datasets, and the need for external validation constrain the generalizability of AI models in real-world clinical settings. Nevertheless, when thoughtfully integrated, AI has the potential to enhance diagnostic accuracy, support early therapeutic decision-making, and reduce unnecessary interventions, ultimately reinforcing its clinical value in the complex differentiation of renal tumors.

The aim of this study was to conduct a systematic review and meta-analysis to assess the pooled diagnostic performance of previously published AI models in differentiating Wilms tumors from non-Wilms tumors using computed tomography imaging.

## Methods

### Literature Search

**Figure 1:**
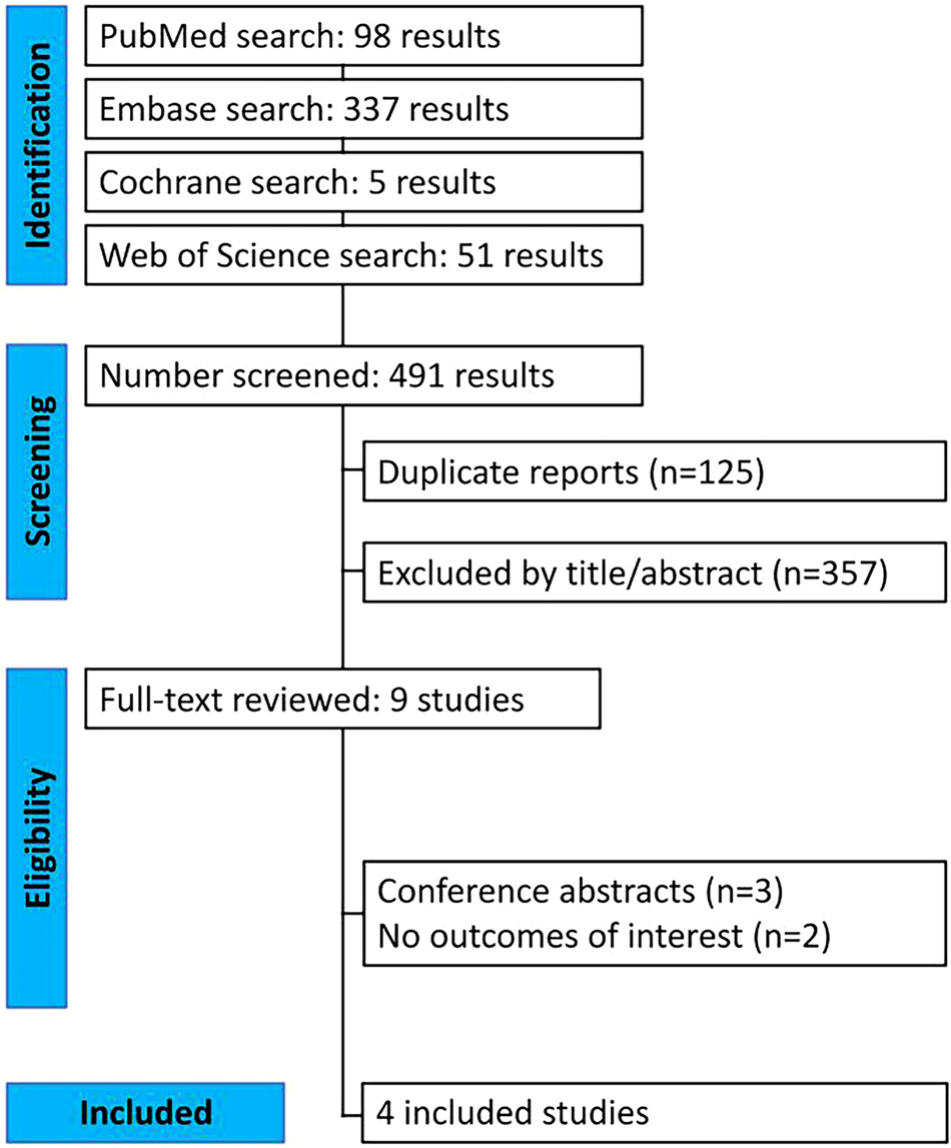
Flowchart of article selection according to PRISMA-DTA 2018.

This research adhered to the Preferred Reporting Items for Systematic Reviews and Meta-Analysis for diagnostic test accuracy (PRISMA-DTA) 2018 protocol (Figure 1)^9^. The final literature search was conducted on December 30, 2024, utilizing Cochrane, Embase, Web of Science and MEDLINE (via PubMed) databases. The search strategy employed was: (“Wilms” OR “nephroblastoma” OR “Wilms tumor” OR “pediatric renal tumor” OR “childhood renal cancer” OR “metanephric blastema” OR “embryonal renal tumor” OR “WT1” OR “PAX2”) AND (“artificial intelligence” OR “machine learning” OR “deep learning” OR “neural networks” OR “convolutional neural network” OR “reinforcement learning” OR “deep belief network” OR “recurrent neural network” OR “feedforward neural network” OR “radiomic*”). The protocol was registered on the PROSPERO platform under the number CRD42024621223.

### Eligibility Criteria

A total of 491 articles were identified during the initial database search. After eliminating duplicates, 366 articles remained. Of these, diagnostic studies that evaluated patients with and without Wilms tumor using computed tomography, under AI analysis, and reported sensitivity (SEN), specificity (SPE), and area under the curve (AUC) data were selected. Studies lacking these data, providing incomplete information, or unavailable in full text were excluded.

### Quality Assessment

Risk of bias was assessed using the Quality Assessment of Diagnostic Accuracy Studies (QUADAS-2) tool^10^. Publication bias was evaluated using funnel plot analysis based on study weight.

### Data Extraction

Three authors participated in data collection: two independently extracted the data, and one reviewed it for accuracy. The following data were assessed: first author’s last name, year and country of publication, AI methods, study design, total number of individuals with and without Wilms tumor, AI objective, kilovoltage peak (kVP), preoperative imaging data phases and seconds, contrast and injection dose, scanning layer thickness, SEN, SPE and AUC.

### Statistical Analysis

Statistical analysis was performed in R software version 4.3.3 using the random-effects model. For univariate analysis, logit transformation with proportional analysis was used, and for bivariate analysis, a summary receiver operating characteristic curve (SROC) was constructed. The I^2^ statistic was used to assess heterogeneity, with values ≥ 40% considered indicative of significant. A leave-one-out sensitivity analysis was conducted when heterogeneity exceeded this threshold to evaluate the influence of individual studies on the overall results.

### Ethical Evaluation

As the studies evaluated in this research are accessible in public databases and portals, the need for an ethical review was exempted. Furthermore, the study did not access or utilize any personal or sensitive data from individuals, since all datasets were anonymized and aggregated. This research complied with applicable ethical standards for the use of published data.

## Results

After full-text screening and data extraction, four studies were included in our meta-analysis (Table 1)^11–14^. The dataset involved 177 patients with Wilms tumors and 62 non-Wilms tumors. Three studies were from China^11,13,14^ and one was from Turkey^12^. All studies had a retrospective, observational design. One dataset^11^ contained a deep learning method and all studies used supervised machine learning algorithms aimed at tumor identification and preoperative support. Table 2 shows the acquisition parameters of the images used in the extraction of radiomic features.

**Table 1:** Baseline characteristics of included studies

Author	Country	Study design	AI methods	Model objective	Wilms/Non-Wilms (test)	Sensitivity/Specificity	AUC
Zhu et al.^11^	China	Retrospective, single-center	ResNet-34 (DL)	Identification and assist in preoperative diagnosis	54/19	56.3%/ 84.2%	0.831
Koska et al.^12^	Turkey	Retrospective, single-center	SVM and RF (ML)	Identification and assist in preoperative diagnosis	29/27	83.0%/ 76.3%	0.940
Song et al.^13^	China	Retrospective, single-center	SVM, LDA, LR, Adaboost, Gaussian process, AE, RE, LR-Lasso, Decision tree and Naïve Bayes (ML)	Identification and assist in preoperative diagnosis	18/5 (CMP) and 13/6 (NP)	60.9%/ 87.5%	0.433 (CMP)/ 0.693 (NP)
Deng et al.^14^	China	Retrospective, single-center	LASSO and LR (ML)	Identification and assist in preoperative diagnosis	25/10	60.0%/ 96.0%	0.792

Abbreviations – SVM: support vector machine; RF: random-forest; DL: deep learning; ML: machine learning; LDA: linear discriminant analysis; LR: logistic regression, AE: Auto-Encoder; CMP: corticomedullary phase; NP: nephrogenic phase; LASSO: Least Absolute Shrinkage and Selection Operator.

**Table 2:** Ct acquisition parameters for images used in radiomic feature extraction to differentiate wilms and non-wilms tumors

Author	kVp	Slice thickness (mm)	Preoperative imaging data phases and seconds	Type of contrast	Amount of IV contrast	Injection dose
Zhu et al.^11^	NA	5 mm	CMP (15–18 s) and NP (45–55 s)	Iohexol	300 mg/ml	1.1–1.6 ml/kg
Koska et al.^12^	NA	1.25, 2, 2.5, 3 and 5 mm	Portal venous (NA)	Iodinated non-ionic	NA	2–3 mL/s
Song et al.^13^	70, 80, 100 and 120 kV	3 and 5 mm	CMP (15–30 s) and NP (60–90 s)	Ioversol	350 mg/mL	1.5 mL/kg
Deng et al.^14^	NA	5.0 mm	CMP (15–30 s) and NP (55–65 s)	Iodine	320 mg/mL	1.0–2.0 mL/kg

Abbreviations – NA: non-available; CMP: corticomedullary phase; NP: nephrogenic phase.

### Combined Analysis

**Figure 2:**
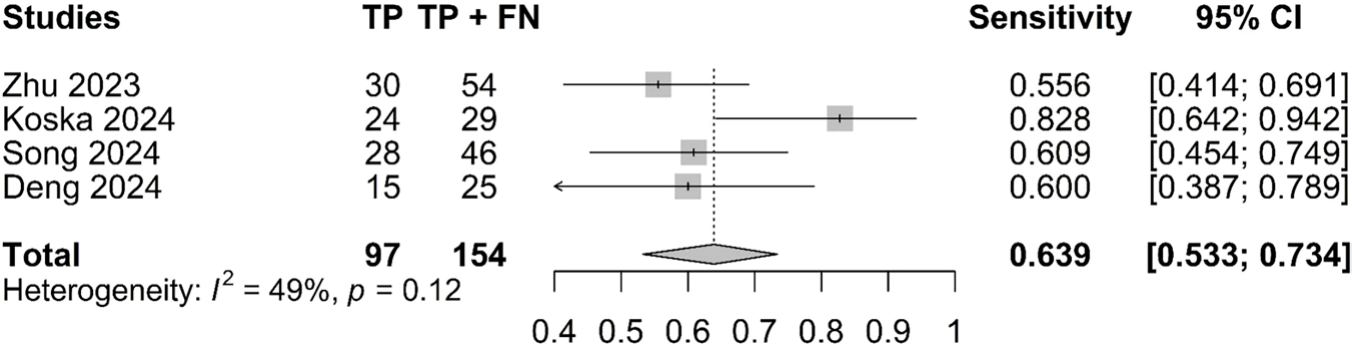
Forest plot for sensitivity of the pooled model.

**Figure 3:**
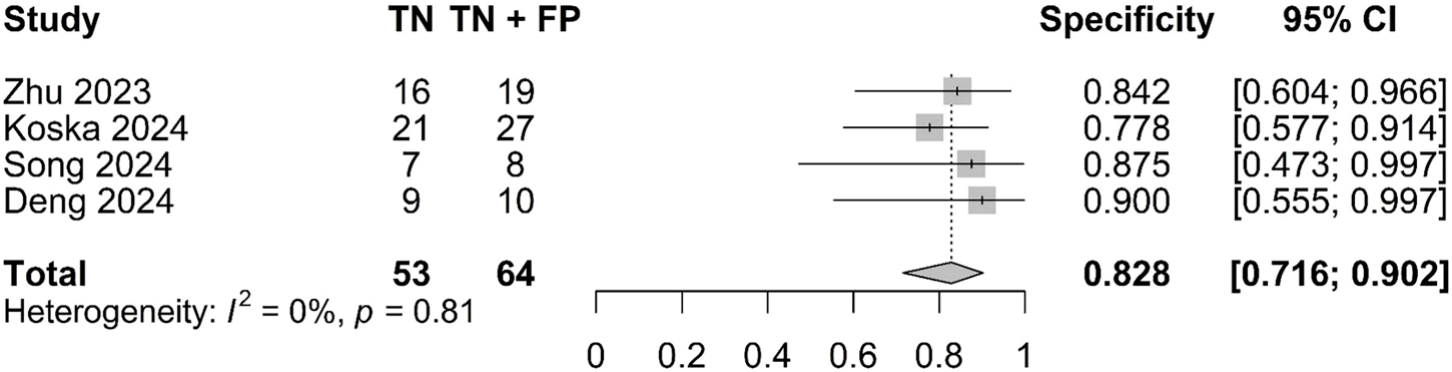
Forest plot for specificity of the pooled model.

**Figure 4:**
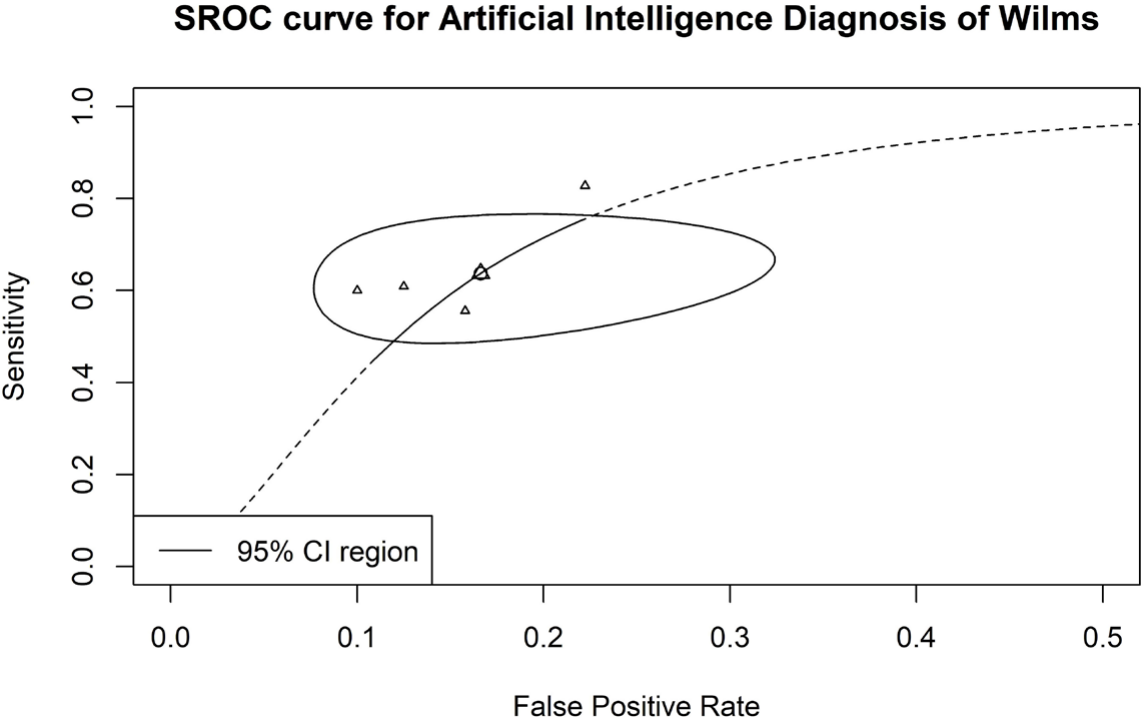
SROC curve for AI diagnosis of Wilms versus non-Wilms tumors.

A forest plot indicated a SEN of 63.9% (95% CI: 0.533–0.734; I^2^ = 49%; Figure 2), SPE of 82.8% (95% CI: 0.716–0.902; I^2^ = 0%; Figure 3) and an AUC of 0.831 (95% CI: 0.607–0.883; Figure 4). Given the observed heterogeneity in SEN, a sensitivity analysis was performed using the leave-one-out method. Notably, when the study by Koska et al.^12^ was excluded, the pooled sensitivity decreased substantially (SEN: 58.0%; 95% CI: 0.50–0.79), and heterogeneity was eliminated (I^2^ = 0%).

### Appraisal of Quality and Scrutiny of Publication Bias

Our risk of bias assessment (Chart 1) showed that all studies^11–14^ had a low risk. When the funnel plot was assessed, it showed the studies were distributed bilaterally, showing no notable asymmetry.

**Chart 1:** Risk of bias analysis

Author	Risk of Bias				Applicability Concerns		
	Patient selection	Index Test	Reference Standard	Flow and timing	Patient selection	Index Test	Reference Standard
Zhu et al.^11^	Low risk	Low risk	Low risk	Low risk	Low risk	Low risk	Low risk
Koska et al.^12^	Low risk	Low risk	Low risk	Low risk	Low risk	Low risk	Low risk
Song et al.^13^	Low risk	Low risk	Low risk	Low risk	Low risk	Low risk	Low risk
Deng et al.^14^	Low risk	Low risk	Low risk	Low risk	Low risk	Low risk	Low risk

## Discussion

This is the first systematic review and meta-analysis focused on the application of AI in pediatric renal tumors. It included more than 200 patients and showed a good diagnostic performance of AI models, although with some heterogeneity in sensitivity findings. Unfortunately, even with the most comprehensive search possible, few studies were found on the subject, concentrated only in Asian countries. However, this work represents a first effort to draw attention to this topic, which could revolutionize the propaedeutics of pediatric kidney tumors.

**Figure 5:**
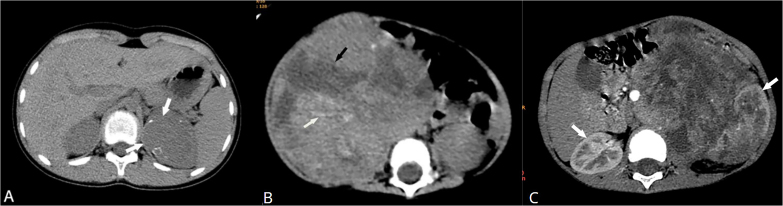
Representative CT images of pediatric renal tumors: A – clear cell carcinoma, B – malignant rhabdoid tumor, C – clear cell sarcoma. Images adapted from Zhu et al.^18^.

While Wilms tumor is the most common pediatric renal malignancy, its incidence varies markedly across age groups. This subtype accounts for 58% of kidney tumors in children under 7 months of age, more than 90% of cases in those aged 1–9 years, and between 54–67% among children aged 10–14 years^15,16,17^. Given its significantly more favorable prognosis, the current challenge is to differentiate Wilms tumor from other tumors such as renal cell carcinoma, malignant rhabdomyoma tumor of the kidney, clear cell sarcoma of the kidney, and congenital mesoblastic nephroma, which collectively categorized into non-Wilms tumors^1^, as shown in Figure 5.

This article is licensed under a Creative Commons Attribution 4.0 International License, which permits use, sharing, adaptation, distribution, and reproduction in any medium or format, provided appropriate credit is given to the original authors and source. Permission for reuse of these images was granted by BMC Medical Imaging.

The difficulties in clinically distinguishing between tumor subtypes have led to divergent treatment protocols. Currently, the Children’s Oncology Group, based mainly in North America, advocates for initial tumor resection followed by the definition of the chemotherapy protocol, which would reduce the use of inappropriate medications and their consequent side effects^19^. On the other hand, the International Society of Paediatric Oncology, which has greater influence in European centers, recommends preoperative chemotherapy to facilitate surgery, reduce the risk of tumor rupture, and minimize the need for subsequent radiotherapy^20^.

Reconciling these approaches may be possible through enhanced non-invasive diagnostic strategies, such as AI-assisted CT interpretation. However, radiologists currently achieve a modest 59.2% diagnostic accuracy in subtype differentiation^13^. Even histology-based chemotherapy decisions after biopsy yield only 86% accuracy, underscoring the need for better preoperative tools^21^.

In view of the above, the application of AI to CT imaging emerges as a valuable adjunctive strategy to improve diagnostic accuracy in pediatric renal tumors, particularly Wilms tumor. Despite the promising overall accuracy of 70.1% observed in this meta-analysis, outperforming senior radiologists and approaching biopsy-level accuracy, this result must be interpreted within the context of sample limitations. Studies in this domain are retrospective, single-center, and based on small cohorts, which limits generalizability and introduces heterogeneity in imaging acquisition protocols and labeling accuracy^11–14^. Additionally, interobserver variability in histopathological confirmation of Wilms tumor subtypes remains a confounding factor, although recent advances in AI-driven histological classification offer hope for reducing diagnostic inconsistency^22^.

Furthermore, studies utilizing AI techniques—such as machine learning and deep learning—in CT for the classification of renal tumors, including renal cell carcinoma (RCC) and angiomyolipoma (AML), have demonstrated higher diagnostic performance compared to our meta-analysis. For instance, Yao et al.^23^ developed a multichannel deep learning model based on unenhanced CT images, reporting AUC values of 0.966 and 0.898 for internal and external validation, respectively, in differentiating fat-poor AML from RCC. Similarly, Feng et al.^24^ employed machine learning with quantitative texture analysis to distinguish small (≤4 cm) AML without visible fat from RCC, achieving an accuracy of 93.9%. Cui et al.^25^ also demonstrated high discriminative performance using support vector machines and texture features, with AUCs reaching up to 0.97 in differentiating AML from various RCC subtypes. In a multicenter study, Toda et al.^26^ developed a fully automated deep learning algorithm for the detection of small RCCs on contrast-enhanced CT, achieving accuracy rates of 88.3% and 87.5% on internal and external datasets, respectively.

However, Zhou et al.^27^ reported that a three-phase CT-based machine learning model yielded an accuracy of up to 86%, effectively complementing radiologists’ performance in distinguishing RCCs from benign renal tumors. Variability in AI model performance is also evident when differentiating Wilms tumors from non-Wilms renal tumors, such as clear cell sarcoma and rhabdoid tumor, which often overlap radiologically. Song et al.^13^ demonstrated that while machine learning models based on CT texture features achieved reasonable performance (AUC up to 0.79), their sensitivity remained modest, indicating a risk of false negatives and underdiagnosis in complex cases. The study emphasized the need for standardized imaging datasets and inclusion of multimodal clinical features to refine classification performance. Furthermore, comparative analyses across imaging modalities suggest that while CT offers spatial resolution and tumor characterization, its efficacy is maximized when interpreted in conjunction with ultrasonography and MRI, especially in atypical or cystic tumor presentations^28^. However, the method would still need to be standardized before more extensive tests can be carried out. While the studies by Zhu et al.^11^, Deng et al.^14^, and Song et al.^13^ used the corticomedullary and nephrogenic phases of the contrast, Koska et al.^12^ seem to have obtained better results by relying only on the portal venous phase. Standardization of radiomics extraction processes across studies is critical for reducing inter-study variability and enhancing external validity^29^.

Moreover, model performance can vary significantly depending on both the machine learning method and the process used to select the radiomic features to be analyzed^30,31^. The articles included in this review align with the broader literature, indicating that less complex techniques such as logistic regression models can generate good results and increase the generalization of the work^13,14^.

Integrating imaging analysis with clinical and laboratory parameters appeared to enhance model sensitivity in the analyzed studies^12^ and has also been proved useful in adult kidney tumors^23^. This proposal is interesting but must be evaluated for cost-effectiveness, as incorporating more features into the model generally increase the computing power required.

Some factors that could not be assessed in the study by Koska et al.^12^ are already known to be of good clinical value in helping differentiate between pediatric kidney tumors, such as urinary catecholamines, which are present in 90% of neuroblastoma cases^32^. Other factors that probably have a good individual predictive value and deserve further study are the presence of tumor thrombus, encasement of vessels, and presence of calcification^30,31,33^.

Future applications of AI in the diagnosis of Wilms tumor should focus on three main directions: (1) the integration of radiogenomic markers to support personalized therapy planning, (2) the development of multi-institutional collaborative databases to overcome data scarcity and reduce algorithmic bias, and (3) the use of real-time AI assistance during radiological evaluation to support clinical decision-making. Moreover, combining AI analysis with circulating tumor DNA (ctDNA) profiling has been suggested as a novel approach to enhance early diagnostic precision and potentially guide neoadjuvant therapy without the need for immediate invasive biopsy^34^. These strategies collectively signal a paradigm shift toward minimally invasive, high-precision diagnostics in pediatric oncology. Furthermore, evaluating the integration of imaging and clinical characteristics from a cost-effectiveness perspective seem to be the next logical step, preceding larger-scale multicenter studies. Once this effectiveness is established, subsequent research should involve a sample that is ethnically diverse and includes centers with different tomographic devices of different resolutions, as well as multiple laboratory kits, if these characteristics are retained as important in the final model^35^.

However, regulatory and implementation challenges remain significant, particularly in developing countries where healthcare infrastructure may be limited. These challenges include the absence of comprehensive and standardized regulatory frameworks for AI in medicine, limited availability of trained personnel for AI deployment and maintenance, and limited financial resources to support technology acquisition and integration. Additionally, data privacy concerns, ethical considerations, and the need for culturally and regionally adapted AI models complicate widespread adoption. Addressing these barriers will require coordinated efforts involving policymakers, healthcare providers, and technology developers to establish clear guidelines, invest in workforce training, and ensure equitable access.

Among the limitations of this review, it is worth highlighting the number of studies included, their single-center nature, and the number of patients as important drawbacks. Additionally, the absence of standardized imaging acquisition protocols across studies leads to variability in image quality, resolution, and contrast settings, which can significantly affect the performance and reproducibility of AI algorithms. Moreover, the considerable heterogeneity in machine learning methodologies—including differences in model architectures, feature extraction techniques, training datasets, and validation strategies—further complicates direct comparisons between studies. This methodological diversity limits the ability to generalize findings, hinders meta-analytic synthesis, and poses challenges for clinical translation, underscoring the urgent need for consensus on imaging standards and harmonized AI development frameworks in this field.

Nevertheless, our research demonstrated that AI methods can be an important auxiliary tool when applied to CT to differentiate patients with Wilms and non-Wilms tumors with good sensitivity and high specificity. Further studies involving larger, multicenter populations are needed so that AI can be increasingly improved and used by several countries around the world.

## Conclusion

This systematic review and meta-analysis constitute an important step in exploring the potential of AI in differentiating Wilms tumors from non-Wilms tumors. Despite limitations such as study heterogeneity, single-center designs, and small sample sizes, the results highlight AI’s promising diagnostic accuracy, surpassing radiologist performance and approaching biopsy-level results without the associated risks. However, the standardization of imaging acquisition protocols, radiomics processes, and integration with clinical data is crucial for broader application. Reinforcing the need for standardized imaging acquisition protocols would further emphasize future research priorities, particularly regarding model reproducibility and generalizability. Future research should focus on validating these models in diverse populations and evaluating cost-effectiveness to pave the way for more accessible, precise diagnostic tools in pediatric oncology.

## Data Availability

The dataset supporting the findings of this study is not publicly available but can be obtained from the corresponding author upon reasonable request.
