# Supplementary material for: Artificial Intelligence computed tomography models for the discrimination of Wilms versus non-Wilms tumors: systematic review and meta-analysis
Source: J Bras Nefrol. 2025 Oct 10;48(1):e20250010. doi: 10.1590/2175-8239-JBN-2025-0010en (PMC12513725; doi:10.1590/2175-8239-JBN-2025-0010en)
Supplement: Translation

# Translation: Modelos de tomografia computadorizada baseados em inteligência artificial para a distinção entre tumores de Wilms e não-Wilms: revisão sistemática e meta-análise

**DOI:** 10.1590/2175-8239-JBN-2025-0010pt

**Authors:** Helvécio Neves Feitosa, Rian Vilar Lima, João Filipe Cavalcante Uchoa Furtado, Victor Arthur Ohannesian, Eduardo Correia Eulálio, Pedro Vianna Caldas Ribeiro, Lucas Macêdo Aurélio Paiva, Isabela Diógenes Feitosa, Geraldo Bezerra da Silva

**Affiliations:** 1 Universidade de Fortaleza, Escola de Medicina, Fortaleza, CE, Brasil.; 2 Faculdade de Ciências da Saúde Albert Einstein, Escola de Medicina, São Paulo, SP, Brasil.; 3 Universidade de Fortaleza, Escola de Medicina, Programas de Pós-Graduação em Saúde Pública e Ciências Médicas, Fortaleza, CE, Brasil.

**Keywords:** Inteligência Artificial, Algoritmos de Aprendizado de Máquina, Meta-Análise, Tumor de Wilms

## Abstract

**Objetivo:**

Realizar revisão sistemática e meta-análise para avaliar a eficácia de modelos de inteligência artificial (IA) destinados a identificar tumores de Wilms em tomografias computadorizadas (TC).

**Métodos:**

Realizou-se uma busca nas bases de dados *MEDLINE, Embase, Web of Science e Cochrane,* conforme as diretrizes PRISMA (*Preferred Reporting Items for Systematic Reviews and Meta-Analyses*) 2020. Foram incluídos estudos diagnósticos utilizando TC baseada em IA para diagnosticar tumores de Wilms, desde que relatassem sensibilidade, especificidade e AUC. Estudos com dados incompletos ou ausência de texto completo foram excluídos. A análise estatística foi realizada no R (v4.3.3) utilizando modelo de efeitos aleatórios, com transformação logit para análise univariada e construção de curva SROC para análise bivariada. A heterogeneidade (I^2^ ≥ 40%) foi avaliada e explorada por análise de sensibilidade.

**Resultados:**

Foram incluídos quatro estudos (três da China e um da Turquia) com 177 pacientes com tumores de Wilms e 62 sem tumores de Wilms. A análise combinada de todos os modelos demonstrou sensibilidade de 63,9% (IC95%: 0,533–0,734), especificidade de 82,8% (IC95%: 0,716–0,902) e área sob a curva (AUC) de 0,831 (IC95%: 0,607–0,883).

**Conclusão:**

Este estudo demonstrou que modelos de IA exibem sensibilidade moderada e alta especificidade para identificar tumores de Wilms na TC, com uma AUC geral de 0,831. Esses resultados ressaltam o potencial promissor da IA como ferramenta de apoio no diagnóstico por imagem, embora o número limitado de estudos e a notável heterogeneidade metodológica justifiquem uma interpretação cautelosa e reforcem a necessidade de validação em populações maiores e mais representativas.

## Introdução

O tumor de Wilms, também denominado nefroblastoma, é a forma mais comum de câncer renal em crianças, representando aproximadamente 95% dos tumores renais pediátricos^1^. Essa malignidade está associada a mutações em genes críticos, como o *WT1*, e alterações epigenéticas em *loci* específicos, como o 11p15^2^. Embora, em geral, apresente um excelente prognóstico, com taxas de cura superiores a 90% em casos localizados, a identificação de fatores genéticos e moleculares permanece essencial para o tratamento personalizado^3^. O manejo clínico se baseia em um esforço colaborativo entre oncologistas pediátricos, cirurgiões e patologistas, que aplicam protocolos específicos para estratificar o risco de cada paciente^4^.

A aplicação de inteligência artificial (IA) demonstra ser bastante promissora no diagnóstico e manejo de tumores renais, incluindo o tumor de Wilms^5^. Algoritmos de *deep learning* podem analisar imagens de tomografia computadorizada com alta precisão, detectando características sutis que podem passar despercebidas por observadores humanos^6^. Além disso, essas ferramentas auxiliam na distinção entre massas renais benignas e malignas, otimizando a tomada de decisões clínicas e reduzindo erros diagnósticos^7^. A integração de dados radiológicos e genômicos por meio da IA oferece *insights* detalhados sobre a evolução e o comportamento tumoral, possibilitando a personalização das intervenções terapêuticas^8^.

Apesar de seu potencial promissor, a aplicação clínica de IA na diferenciação de tumores renais ainda enfrenta desafios significativos. A sobreposição de características radiológicas entre diversas neoplasias renais — como tumor de Wilms, nefroma mesoblástico congênito e outras massas renais pediátricas — pode limitar a especificidade do diagnóstico, mesmo para algoritmos avançados. Além disso, a variabilidade nos protocolos de imagem, o acesso limitado a grandes conjuntos de dados e a necessidade de validação externa restringem a generalização dos modelos de IA em cenários clínicos reais. No entanto, quando cuidadosamente integrada, a IA tem o potencial de aumentar a precisão do diagnóstico, apoiar a tomada de decisões terapêuticas precoces e reduzir intervenções desnecessárias, reforçando, em última instância, seu valor clínico na complexa diferenciação de tumores renais.

O objetivo deste estudo foi realizar uma revisão sistemática e meta-análise para avaliar o desempenho diagnóstico combinado de modelos de IA publicados anteriormente na diferenciação entre tumores de Wilms e tumores não-Wilms utilizando imagens de tomografia computadorizada.

## Métodos

### Pesquisa Bibliográfica

Esta pesquisa seguiu o protocolo *Preferred Reporting Items for Systematic Reviews and Meta-Analysis for diagnostic test accuracy* (PRISMA-DTA) 2018 (Figura 1)^9^. A busca final na literatura foi realizada em 30 de dezembro de 2024, utilizando as bases de dados *Cochrane*, *Embase*, *Web of Science* e MEDLINE (via *PubMed*). A estratégia de busca empregada foi: (“*Wilms*” OR “*nephroblastoma*” OR “*Wilms tumor*” OR “*pediatric renal tumor*” OR “*childhood renal cancer*” OR “*metanephric blastema*” OR “*embryonal renal tumor*” OR “*WT1*” OR “*PAX2*”) AND (“*artificial intelligence*” OR “*machine learning*” OR “*deep learning*” OR “*neural networks*” OR “*convolutional neural network*” OR “*reinforcement learning*” OR “*deep belief network*” OR “*recurrent neural network*” OR “*feedforward neural network*” OR “radiomic*”). O protocolo foi registrado na plataforma PROSPERO sob o número CRD42024621223.

**Figura 1:** Fluxograma de seleção de artigos conforme o PRISMA-DTA 2018.

### Critérios de Elegibilidade

Um total de 491 artigos foi identificado durante a busca inicial na base de dados. Após a eliminação de duplicatas, foram mantidos 366 artigos. Dentre estes, foram selecionados estudos diagnósticos que avaliaram pacientes com e sem tumor de Wilms por meio de tomografia computadorizada, sob análise de IA, e que relataram dados de sensibilidade (SEN), especificidade (ESP) e área sob a curva (AUC). Foram excluídos estudos que não apresentavam esses dados, forneciam informações incompletas ou não estavam disponíveis na íntegra.

### Avaliação da Qualidade

O risco de viés foi avaliado utilizando a ferramenta *Quality Assessment of Diagnostic Accuracy Studies* (QUADAS-2)^10^. O viés de publicação foi determinado por meio de análise de gráfico de funil com base no peso do estudo.

### Extração de Dados

Três autores participaram da coleta de dados: dois extraíram os dados de forma independente e um revisou-os para garantir a precisão. Foram avaliados os seguintes dados: sobrenome do primeiro autor, ano e país de publicação, métodos de IA, desenho do estudo, número total de indivíduos com e sem tumor de Wilms, objetivo da IA, pico de quilovoltagem (kVP), fases e segundos dos dados de imagem pré-operatória, contraste e dose de injeção, espessura da camada de varredura, SEN, ESP e AUC.

### Análise Estatística

A análise estatística foi realizada no software R, versão 4.3.3, utilizando o modelo de efeitos aleatórios. Para a análise univariada, empregou-se a transformação *logit* com análise proporcional e, para a análise bivariada, construiu-se uma curva SROC (*Summary Receiver Operating Characteristic*). A estatística I^2^ foi utilizada para avaliar a heterogeneidade, com valores ≥ 40% considerados indicativos de significância. Quando a heterogeneidade excedeu esse limiar, realizou-se uma análise de sensibilidade *leave-one-out* para avaliar a influência de estudos individuais nos resultados gerais.

### Avaliação Ética

Como os estudos avaliados nesta pesquisa estão disponíveis em bancos de dados e portais públicos, a necessidade de uma revisão ética foi dispensada. Além disso, o estudo não acessou nem utilizou quaisquer dados pessoais ou sensíveis dos indivíduos, uma vez que todos os conjuntos de dados foram anonimizados e agregados. Esta pesquisa cumpriu as normas éticas aplicáveis ao uso de dados publicados.

## Resultados

Após a triagem do texto completo e a extração dos dados, quatro estudos foram incluídos em nossa meta-análise (Tabela 1)^11–14^. O conjunto de dados envolveu 177 pacientes com tumores de Wilms e 62 com tumores não-Wilms. Três estudos foram realizados na China^11,13,14^ e um na Turquia^12^. Todos os estudos apresentaram um desenho retrospectivo e observacional. Um dos conjuntos de dados^11^ continha um método de *deep learning* e todos os estudos utilizaram algoritmos de *machine learning* supervisionado, com o objetivo de identificar tumores e fornecer suporte pré-operatório. A Tabela 2 apresenta os parâmetros de aquisição das imagens utilizadas na extração das características radiômicas.

**Tabela 1:** Características basais dos estudos incluídos

Autor	País	Desenho do estudo	Métodos de IA	Objetivo do modelo	Wilms/Não-Wilms (teste)	Sensibilidade/Especificidade	AUC
Zhu et al.^11^	China	Retrospectivo, centro único	ResNet-34 (DL)	Identificação e auxílio no diagnóstico pré-operatório	54/19	56,3%/ 84,2%	0,831
Koska et al.^12^	Turquia	Retrospectivo, centro único	SVM e RF (ML)	Identificação e auxílio no diagnóstico pré-operatório	29/27	83,0%/ 76,3%	0,940
Song et al.^13^	China	Retrospectivo, centro único	SVM, LDA, RL, *Adaboost*, Processo Gaussiano, AE, RE, LR-Lasso, Árvore de Decisão e *Naïve Bayes* (ML)	Identificação e auxílio no diagnóstico pré-operatório	18/5 (FCM) e 13/6 (FN)	60,9%/ 87,5%	0,433 (FCM)/ 0,693 (FN)
Deng et al.^14^	China	Retrospectivo, centro único	LASSO e RL (ML)	Identificação e auxílio no diagnóstico pré-operatório	25/10	60,0%/ 96,0%	0,792

Abreviaturas – SVM: máquina de vetores de suporte; RF: *random-forest*; DL: *deep learning*; ML: *machine learning*; LDA: análise discriminante linear; RL: regressão logística, AE: *Auto-Encoder*; FCM: fase corticomedular; FN: fase nefrogênica; LASSO: Operador de Seleção e Menor Contração Absoluta.

**Tabela 2:** Parâmetros de aquisição de tc para imagens utilizadas na extração de características radiômicas para diferenciar tumores wilms e não-wilms

Autor	kVp	Espessura da camada (mm)	Fases e segundos dos dados de imagem pré-operatória	Tipo de contraste	Quantidade de contraste IV	Dose da injeção
Zhu et al.^11^	NA	5,0 mm	FCM (15–18 s) e FN (45–55 s)	Iohexol	300 mg/ml	1,1–1,6 ml/kg
Koska et al.^12^	NA	1,25; 2; 2,5; 3 e 5 mm	Veia porta (NA)	Iodado não iônico	NA	2–3 mL/s
Song et al.^13^	70, 80, 100 e 120 kV	3 e 5 mm	FCM (15–30 s) e FN (60–90 s)	Ioversol	350 mg/mL	1,5 mL/kg
Deng et al.^14^	NA	5,0 mm	FCM (15–30 s) e FN (55–65 s)	Iodo	320 mg/mL	1,0–2,0 mL/kg

Abreviaturas – NA: não disponível; FCM: fase corticomedular; FN: fase nefrogênica.

### Análise Combinada

Um *forest plot* indicou uma SEN de 63,9% (IC95%: 0,533–0,734; I^2^ = 49%; Figura 2), ESP de 82,8% (IC95%: 0,716–0,902; I^2^ = 0%; Figura 3) e uma AUC de 0,831 (IC95%: 0,607–0,883; Figura 4). Dada a heterogeneidade observada na SEN, foi realizada uma análise de sensibilidade utilizando o método *leave-one-out*. Notavelmente, quando o estudo de Koska et al.^12^ foi excluído, a sensibilidade agrupada apresentou uma redução substancial (SEN: 58,0%; IC95%: 0,50–0,79) e a heterogeneidade foi eliminada (I^2^ = 0%).

**Figura 2:** *Forest plot* para a sensibilidade do modelo agrupado.

**Figura 3:** *Forest plot* para a especificidade do modelo agrupado.

**Figura 4:** Curva SROC para diagnóstico por IA de tumores de Wilms *versus* tumores não-Wilms.

### Avaliação da Qualidade e Análise do Viés de Publicação

Nossa avaliação do risco de viés (Quadro 1) demonstrou que todos os estudos^11–14^ apresentaram baixo risco. Ao avaliar o gráfico de funil, observou-se que os estudos estavam distribuídos bilateralmente, sem apresentar assimetria notável.

**Quadro 1:** Análise do risco de viés

Autor	Risco de Viés				Questões de aplicabilidade		
	Seleção de pacientes	Teste de Índice	Padrão de Referência	Fluxo e tempo	Seleção de pacientes	Teste de Índice	Padrão de Referência
Zhu et al.^11^	Baixo risco	Baixo risco	Baixo risco	Baixo risco	Baixo risco	Baixo risco	Baixo risco
Koska et al.^12^	Baixo risco	Baixo risco	Baixo risco	Baixo risco	Baixo risco	Baixo risco	Baixo risco
Song et al.^13^	Baixo risco	Baixo risco	Baixo risco	Baixo risco	Baixo risco	Baixo risco	Baixo risco
Deng et al.^14^	Baixo risco	Baixo risco	Baixo risco	Baixo risco	Baixo risco	Baixo risco	Baixo risco

## Discussão

Esta é a primeira revisão sistemática e meta-análise focada na aplicação de IA em tumores renais pediátricos. O estudo incluiu mais de 200 pacientes e demonstrou um bom desempenho diagnóstico dos modelos de IA, embora com alguma heterogeneidade nos achados de sensibilidade. Infelizmente, mesmo com a busca mais abrangente possível, foram encontrados poucos estudos sobre o tema, concentrados somente em países asiáticos. No entanto, este trabalho representa um primeiro esforço para chamar a atenção para este tópico, que pode revolucionar a propedêutica dos tumores renais pediátricos.

Embora o tumor de Wilms seja a neoplasia renal pediátrica mais comum, sua incidência varia significativamente entre as faixas etárias. Esse subtipo é responsável por 58% dos tumores renais em crianças com menos de 7 meses de idade, mais de 90% dos casos naquelas com 1 a 9 anos e entre 54% e 67% entre crianças com 10 a 14 anos^15–17^. Dado seu prognóstico significativamente mais favorável, o desafio atual consiste em diferenciar o tumor de Wilms de outras neoplasias, como carcinoma de células renais, tumor rabdoide maligno do rim, sarcoma de células claras do rim e nefroma mesoblástico congênito, que são coletivamente categorizados como tumores não-Wilms^1^, conforme ilustrado na Figura 5.

**Figura 5:** Imagens representativas de TC de tumores renais pediátricos: A – carcinoma de células claras, B – tumor rabdoide maligno, C – sarcoma de células claras. Imagens adaptadas de Zhu et al.^18^.

Este artigo está licenciado sob a Licença *Creative Commons Attribution*, versão 4.0 Internacional, que permite o uso, compartilhamento, adaptação, distribuição e reprodução em qualquer meio ou formato, desde que os autores originais e a fonte sejam devidamente creditados. A permissão para reutilização dessas imagens foi concedida pela *BMC Medical Imaging*.

As dificuldades em distinguir clinicamente entre os subtipos de tumores têm levado à adoção de protocolos de tratamento divergentes. Atualmente, o *Children’s Oncology Group*, com sede principalmente na América do Norte, defende a ressecção inicial do tumor seguida pela definição do protocolo de quimioterapia, o que reduziria o uso de medicamentos inadequados e seus consequentes efeitos colaterais.^19^. Por outro lado, a *International Society of Paediatric Oncology*, que exerce maior influência nos centros europeus, recomenda a quimioterapia pré-operatória, com o objetivo de facilitar a cirurgia, reduzir o risco de ruptura tumoral e minimizar a necessidade de radioterapia subsequente^20^.

A conciliação dessas abordagens pode ser possível por meio de estratégias diagnósticas não invasivas aprimoradas, como a interpretação de TC assistida por IA. No entanto, os radiologistas atualmente alcançam uma precisão diagnóstica modesta de 59,2% na diferenciação de subtipos^13^. Mesmo as decisões de quimioterapia baseadas na histologia após a biópsia apresentam apenas 86% de precisão, ressaltando a necessidade de ferramentas pré-operatórias mais eficazes^21^.

Diante do exposto, a aplicação de IA às imagens de TC surge como uma estratégia adjuvante valiosa para aprimorar a precisão diagnóstica em tumores renais pediátricos, particularmente o tumor de Wilms. Apesar da precisão geral promissora de 70,1% observada nesta meta-análise, superando radiologistas experientes e aproximando-se da precisão ao nível de biópsia, este resultado deve ser interpretado dentro do contexto das limitações da amostra. Os estudos nessa área são retrospectivos, de centro único e baseados em pequenas coortes, o que limita a generalização e introduz heterogeneidade nos protocolos de aquisição de imagens e na precisão da rotulagem^11–14^. Além disso, a variabilidade entre observadores na confirmação histopatológica dos subtipos de tumor de Wilms permanece como um fator de confusão, embora os recentes avanços na classificação histológica assistida por IA ofereçam perspectiva de redução da inconsistência diagnóstica^22^.

Além disso, estudos que utilizam técnicas de IA — como *machine learning* e *deep learning* — em TC para a classificação de tumores renais, incluindo carcinoma de células renais (CCR) e angiomiolipoma (AML), demonstraram um desempenho diagnóstico superior em comparação à nossa meta-análise. Por exemplo, Yao et al.^23^ desenvolveram um modelo de *deep learning* multicanal baseado em imagens de TC sem contraste, relatando valores de AUC de 0,966 e 0,898 para validação interna e externa, respectivamente, na diferenciação entre AML pobre em gordura e CCR. De forma semelhante, Feng et al.^24^ empregaram *machine learning* com análise quantitativa de textura para distinguir entre AML pequenos (≤4 cm) sem gordura visível +e CCR, alcançando uma precisão de 93,9%. Cui et al.^25^ também demonstraram alto desempenho discriminatório utilizando máquinas de vetores de suporte e características de textura, com AUCs atingindo até 0,97 na diferenciação entre AML e diversos subtipos de CCR. Em um estudo multicêntrico, Toda et al.^26^ desenvolveram um algoritmo de *deep learning* totalmente automatizado para a detecção de pequenos CCRs em TC com contraste, alcançando taxas de precisão de 88,3% e 87,5% em conjuntos de dados internos e externos, respectivamente.

No entanto, Zhou et al.^27^ relataram que um modelo de *machine learning* baseado em TC trifásica apresentou uma precisão de até 86%, complementando de forma eficaz o desempenho dos radiologistas na distinção entre CCRs e tumores renais benignos. A variabilidade no desempenho do modelo de IA também é evidente na diferenciação entre tumores de Wilms e tumores renais não-Wilms, como o sarcoma de células claras e o tumor rabdoide, que frequentemente apresentam sobreposição radiológica. Song et al.^13^ demonstraram que, embora os modelos de *machine learning* baseados em características de textura da TC tenham alcançado um desempenho razoável (AUC de até 0,79), sua sensibilidade permaneceu modesta, indicando risco de falsos negativos e de subdiagnóstico em casos complexos. O estudo enfatizou a necessidade de conjuntos de dados de imagem padronizados e a inclusão de características clínicas multimodais para refinar o desempenho da classificação. Além disso, análises comparativas entre modalidades de imagem sugerem que, embora a TC ofereça resolução espacial e caracterização do tumor, sua eficácia é maximizada quando interpretada em conjunto com a ultrassonografia e a RM, especialmente em apresentações tumorais atípicas ou císticas^28^. Entretanto, o método ainda precisaria ser padronizado antes que testes mais extensivos possam ser realizados. Enquanto os estudos de Zhu et al.^11^, Deng et al.^14^ e Song et al.^13^ utilizaram as fases corticomedular e nefrogênica do contraste, Koska et al.^12^ parecem ter obtido melhores resultados ao se basearem apenas na fase venosa portal. A padronização dos processos de extração radiômica entre os estudos é fundamental para reduzir a variabilidade entre os estudos e aumentar a validade externa^29^.

Além disso, o desempenho do modelo pode variar significativamente, dependendo tanto do método de *machine learning* quanto do processo utilizado para selecionar as características radiômicas a serem analisadas^30,31^. Os artigos incluídos nesta revisão estão em consonância com a literatura mais ampla, indicando que técnicas menos complexas, como modelos de regressão logística, podem gerar bons resultados e aumentar a capacidade de generalização do trabalho^13,14^.

A integração da análise de imagens com parâmetros clínicos e laboratoriais pareceu aumentar a sensibilidade do modelo nos estudos analisados^12^, tendo-se também revelado útil em tumores renais em adultos^23^. Esta proposta é interessante, mas deve ser avaliada em termos de custo-efetividade, uma vez que a incorporação de mais características no modelo geralmente aumenta a demanda de poder computacional.

Alguns fatores que não puderam ser avaliados no estudo de Koska et al.^12^ já são conhecidos por apresentarem bom valor clínico na diferenciação entre tumores renais pediátricos, como as catecolaminas urinárias, presentes em 90% dos casos de neuroblastoma^32^. Outros fatores que provavelmente têm um bom valor preditivo individual e merecem estudos adicionais incluem a presença de trombos tumorais, o envolvimento de vasos e a presença de calcificação^30,31,33^.

As futuras aplicações da IA no diagnóstico do tumor de Wilms devem se concentrar em três direções principais: (1) a integração de marcadores radiogenômicos para apoiar o planejamento terapêutico personalizado; (2) o desenvolvimento de bancos de dados colaborativos multi-institucionais para superar a escassez de dados e reduzir o viés algorítmico; e (3) o uso de assistência de IA em tempo real durante a avaliação radiológica para apoiar a tomada de decisões clínicas. Além disso, a combinação da análise por IA com o perfil do DNA tumoral circulante (ctDNA) tem sido sugerida como uma nova abordagem para aumentar a precisão diagnóstica precoce e, potencialmente, orientar a terapia neoadjuvante sem a necessidade de biópsia invasiva imediata^34^. Essas estratégias, em conjunto, sinalizam uma mudança de paradigma em direção a diagnósticos minimamente invasivos e de alta precisão na oncologia pediátrica. Além disso, avaliar a integração de características clínicas e de imagem sob a perspectiva da relação custo-benefício parece ser o próximo passo lógico, precedendo estudos multicêntricos em maior escala. Uma vez estabelecida essa eficácia, pesquisas subsequentes deverão envolver uma amostra etnicamente diversificada e incluir centros com diferentes dispositivos tomográficos de diferentes resoluções, bem como múltiplos kits laboratoriais, caso essas características se mantenham relevantes no modelo final^35^.

No entanto, os desafios regulatórios e de implementação continuam sendo significativos, especialmente em países em desenvolvimento, onde a infraestrutura de saúde pode ser limitada. Esses desafios incluem a ausência de marcos regulatórios abrangentes e padronizados para a IA na medicina, a disponibilidade limitada de profissionais treinados para a implantação e manutenção da IA, além de recursos financeiros limitados para apoiar a aquisição e integração da tecnologia. Além disso, questões relacionadas à privacidade de dados, considerações éticas e a necessidade de modelos de IA adaptados cultural e regionalmente complicam a adoção generalizada. Superar essas barreiras exigirá esforços coordenados envolvendo formuladores de políticas, profissionais de saúde e desenvolvedores de tecnologia, a fim de estabelecer diretrizes claras, investir na capacitação da força de trabalho e garantir o acesso equitativo.

Entre as limitações desta revisão, vale destacar o número de estudos incluídos, sua natureza de centro único e o número de pacientes como desvantagens significativas. Além disso, a ausência de protocolos padronizados de aquisição de imagens entre os estudos resulta em variabilidade na qualidade da imagem, resolução e configurações de contraste, o que pode afetar de forma significativa o desempenho e a reprodutibilidade dos algoritmos de IA. Adicionalmente, a considerável heterogeneidade nas metodologias de *machine learning* — incluindo diferenças nas arquiteturas dos modelos, técnicas de extração de características, conjuntos de dados de treinamento e estratégias de validação — dificulta ainda mais as comparações diretas entre os estudos. Essa diversidade metodológica limita a capacidade de generalizar os achados, dificulta a síntese meta-analítica e impõe desafios à tradução clínica, ressaltando a necessidade urgente de um consenso quanto aos padrões de imagem e estruturas harmonizadas de desenvolvimento de IA neste campo.

Não obstante, nossa pesquisa demonstrou que os métodos de IA podem ser uma importante ferramenta auxiliar quando aplicados à TC para diferenciar pacientes com tumores de Wilms e não-Wilms, apresentando boa sensibilidade e alta especificidade. São necessários estudos adicionais envolvendo populações maiores e multicêntricas para que a IA possa ser cada vez mais aprimorada e utilizada por diversos países ao redor do mundo.

## Conclusão

Esta revisão sistemática e meta-análise constituem um passo importante na exploração do potencial da IA na diferenciação entre tumores de Wilms e tumores não-Wilms. Apesar das limitações, tais como a heterogeneidade do estudo, os desenhos de centro único e as pequenas dimensões das amostras, os resultados destacam a promissora precisão diagnóstica da IA, superando o desempenho de radiologistas e aproximando-se dos resultados ao nível de biópsia, sem os riscos associados. No entanto, a padronização dos protocolos de aquisição de imagens, dos processos radiômicos e da integração com dados clínicos é crucial para uma aplicação mais ampla. Reforçar a necessidade de protocolos padronizados de aquisição de imagens destacaria ainda mais as prioridades de pesquisa futuras, particularmente no que se refere à reprodutibilidade e generalização dos modelos. Pesquisas futuras devem se concentrar na validação desses modelos em populações diversas e na avaliação da relação custo-benefício, a fim de abrir caminho para ferramentas diagnósticas mais acessíveis e precisas na oncologia pediátrica.

## Data Availability

O conjunto de dados que sustenta os achados deste estudo não está disponível publicamente, mas pode ser obtido junto ao autor correspondente mediante solicitação razoável.
